# Comparative Expression Analysis of *HSP70, HSP90, IL-4, TNF, KITLG* and *KIT-receptor* Gene between Varicocele-Induced and Non-Varicocele Testes of Dog

**DOI:** 10.22074/ijfs.2017.5020

**Published:** 2017-09-03

**Authors:** Hossein Hassanpour, Amin Bigham Sadegh, Iraj Karimi, Heidar Heidari Khoei, Azarnoush Karimi, Parinaz Edalati Shaarbaf, Tahereh Karimi Shayan

**Affiliations:** 1 Research Institute of Animal Embryo Technology, Shahrekord University, Shahrekord, Iran; 2 Department of Veterinary Surgery and Radiology, Faculty of Veterinary Medicine, Shahrekord University, Shahrekord, Iran; 3 Department of Pathobiology, Faculty of Veterinary Medicine, Shahrekord University, Shahrekord, Iran; 4 Department of Veterinary Medicine, Science and Research Branch, Islamic Azad University, Tehran, Iran

**Keywords:** Varicocele, Dog, Gene Expression

## Abstract

**Background:**

This study was designed to create an experimental varicocele model by a simple surgical procedure in dog with minimum invasion and to investigate the effect of varicocele-induced infertility on the expression of six related genes (*HSP90, HSP70, IL-4, TNF, KITLG* and *KIT receptor*).

**Materials and Methods:**

In this experimental study, the proximal part of the pampini-form plexus of dog testes was partially occluded without abdominal incision which was confirmed by venographic examination. To evaluate varicocele in its acute form, dogs were castrated after 15 days and testes were dissected. Histopathologic evaluation was undertaken and the relative expression of the six genes was assessed by quantitative realtime polymerase chain reaction (PCR).

**Results:**

Microscopic changes showed tubule degeneration. The Johnson score was significantly decreased in the varicocele testes when compared with non-varicocele testes. Expressions of *HSP90, TNF, KITLG* and the *KIT-receptor* gene were significantly downregulated (P=0.029, 0.047, 0.004 and 0.035 respectively) in varicocele-induced testes while *HSP70* was upregulated (P=0.018). IL-4 did not show differential expression (P=0.377).

**Conclusion:**

We conclude that partial occlusion of the proximal part of the pampiniform plexus induces varicocele in the testis of dog. Differential expression of the mentioned genes may be responsible for the pathophysiology of varicocele and related subfertility.

## Introduction

Varicocele is a pathologic dilation of the venous pampiniform plexus in the spermatic cord (1) and is thought to be associated with male infertility. Diagnostic techniques such as scrotal ultrasonography and color Doppler imaging have demonstrated that varicocele may be the cause of 91% of subfertile human cases (2, 3). The pathophysiology of testicular damage in varicocele is not completely understood, however, histopathologic testicular damages due to varicocele are well documented. The effect of varicocele varies, but may often result in a generalized failure of sperm production (from oligozoospermia to complete nonobstructive azoospermia) (4). Varicocele not only affects the normal function and the fertilizing capacity of the sperm, but it also affects the reproductive potential of the haploid male gamete (5). Several studies have suggested varicocele-mediated mechanisms to explain impaired spermatogenesis (6-8). Impaired temperature regulation and reactive oxygen species (ROS) production may lead to DNA damage and progressive apoptosis of testicular cells (9-12). Research at cellular and molecular level, while still in its infancy, may provide additional insights into the varicocele puzzle (8).

The signaling of KIT is well-known for its ability to potentiate cell survival, proliferation and differentiation. The KIT receptor and its ligand, KIT ligand (KITLG), have been widely studied (13). The KIT receptor is a transmembrane protein with tyrosine kinase activity and a member of the type III receptor tyrosine kinase family. Binding of KITLG to KIT leads to the activation of multiple pathways including Src kinase, phospholipase C (PLC)-γ, Janus kinase (JAK)/signal transducers and activators of transcription (STAT), mitogen activated protein (MAP) kinase and phosphatidyl-inositol-3 (PI3)-kinase pathways (14, 15). Dysfunction of KIT signaling thus results in an array of developmental defects in melanogenesis, hematopoiesis, gametogenesis and spermatogenesis (14, 16, 17).

Cytokines are small soluble proteins with a crucial role in the regulation of inflammatory responses. Also, they transmit signals to surrounding cells for the regulation of cell growth and differentiation. They could trigger complex intracellular signaling events that regulate gene expression required for the cellular response (18). A number of studies have reported that KIT expression is regulated by various proinflammatory signals (16, 19). Differential effects are induced by some cytokines depending on the type of the cell system. Among cytokines, interleukin 1 (IL-1), tumour necrosis factor (TNF), IL-4, granulocyte- macrophage colony stimulating factor fibroblast growth factor (FGF) and IL-10 have been reported to change KIT synthesis (20, 21). However, the effects of cytokines and KIT signaling in the inflammatory process of varicocele are predicatable.

The heat shock proteins (HSPs), a family of endogenous, protective proteins, are located in the cytoplasm and nucleus (e.g. HSP70 and HSP90 respectively) to maintain normal cellular function. ROS, cytotoxic lysosomal enzymes and cytoskeletal alterations are able to activate HSP expression. HSPs, in turn, suppress pro-inflammatory cytokines, reduce oxidative bursts, repair ion channels, protect against the toxic effect of nitric oxide, modulate immune-mediated injuries and prevent apoptosis. The function of HSP and its dependant factors in inflammation provides a basis for its possible involvement in the pathophysiology of varicocele. Indeed, the presence of many HSPs in varicocele has been confirmed by previous studies (22, 23). In the present study, we therefore aimed to investigate histopathologic changes in the varicocele testis and whether same changes can be identified in non-varicocele testis. Variation in the expression of *HSP90, HSP70, IL-4, TNF, KITLG* and the *KIT-receptor* gene, and their potential contribution to varicocelemediated infertility is discussed.

## Materials and Methods

In this experimental study, six adult male crossbred dogs (2-4 years old) with normal quality and approximately 30 kg weight were used in this experiment. They were cared for in the Faculty of Shahrekord Veterinary Medicine and housed in pens with ample run. Commercial food was provided twice a day and the dogs had free access to water. Anti-parasitic drugs were administrated to all dogs (mebendozole, 22 mg/kg, orally for 6 days and praziquantel, 5 mg/kg, orally once). All animals were maintained according to the guidelines of Animal Care and Use Committee of the Faculty of Shahrekord Veterinary Medicine.

### Experimental varicocele induction in dog

To induce experimental varicocele by surgery, the inguinal canal region of dogs was prepared aseptically for operation. Dogs were sedated with 2% acepromazine (0.2 mg/kg) and anesthetized by ketamine and then maintained with 2% halothane. An incision was made in the skin of the inguinal canal region while animals were in the dorsal recumbent position. Spermatic cord was exposed and tunica vaginalis was incised to expose the pampiniform plexus. To make a partial occlusion and congestion in the pampiniform plexus, a piece of silicone tube (INWAY^®^ Suprapubic Catheter, pfm Medical Co., Germany) of 1 cm long was longitudinally incised and opened, and then proximal part of the pampiniform plexus was cited in it. To prevent the movement of the tube, three interrupted sutures were applied by 2.0 absorbable suture material and the skin was sutured by non-absorbable suture material. Dogs were kept for 2 weeks and the diameter of the testes were examined and recorded. On the 15^th^ postoperative day, the animals were anesthetized, their spermatic cord was incised and 2 milliliters of iohexol contrast media (iodixanol, Visipaque 320, GE Healthcare, Canada) was injected in the testicular vein and radiographs were taken immediately from the injected area. This venography was done to confirm congestion and dilation of the venous pampiniform plexus in the spermatic cord of varicocele-induced testis. Finally, non-varicocele (left) and varicoceleinduced (right) testes were dissected by castration of dogs. This was undertaken after two weeks to evaluate varicocele in its acute form (short time) as observed in many adult men (24). Half of each testis was immediately frozen in liquid nitrogen and stored at -70°C for subsequent RNA and expression analyses. Another half was fixed in formalin solution followed by embedding in glycol methacrylate for histopathologic evaluation.

### Histopathologic evaluation

Histopathologic evaluation of the induced varicocele model was carried out by hematoxylin and eosin staining in the non-varicocele and varicoceleinduced testes. To examine spermatogenic activity, spermatogenesis was categorized by using the Johnson score (25). A grade from 1 to 10 for each tubule cross section was provided according to the following criteria: i. No germ cells and no Sertoli cells present, ii. No germ cells but only Sertoli cells present, iii. Only spermatogonia present, iv. Only a few spermatocytes present, v. No spermatozoa or spermatids but many spermatocytes present, vi. Only a few spermatids present, vii. No spermatozoa but many spermatids present, viii. Only a few spermatozoa present, ix. Many spermatozoa present and disorganized spermatogenesis, and x. Complete spermatogenesis and perfect tubules.

### RNA extraction and cDNA synthesis

Total RNA from left (non-varicocele) and right (varicocele) testes was extracted using the Rimazol reagent (Sinaclon Bioscience, Iran) and then homogenized (Sinaclon Bioscience, Iran). The quantity of extracted RNA was then measured by spectrophotometry. Only RNA samples with an absorbance ratio (A260/280) of ≥1.9 was used for synthesis of cDNA (26). Gel agarose (2%) electrophoresis (stained with ethidium bromide) was applied to analyze the quality of extracted RNA. The cDNA was produced from total RNA using M-MLV reverse transcriptase (Sinaclon Bioscience, Iran) according to the protocol of a previous study (27). To denature residual RNA in the cDNA mix, the sample was heated at 75°C for 15 minutes and subsequently stored at -20°C.

### Quantitative real time polymerase chain reaction analysis

The expression levels of *HSP90, HSP70, IL-4, TNF, KITLG,* the *KIT-receptor* gene and GAPDH (encoding glyceraldehyde-3-phosphate dehydrogenase) transcripts were determined by reverse transcriptase quantitative polymerase chain reaction (RT-qPCR) using the EvaGreen chemistry (Sinaclon Bioscience, Iran). To normalise the input load of cDNA between samples, GAPDH levels were used as an internal control which was confirmed as a strong reference gene using Normfinder v20 (Skejby Sygehus, Denmark) in this experiment. Specific primers were designed based on mRNA sequences with Primer-Blast (www.ncbi.nlm.nih.gov/tools/ primer-blast/). All primer sequences are given in Table 1. PCR reactions were carried out in a real-time PCR cycler (Rotor Gene Q 6000, Qiagen, USA) in triplicate for each sample of testis. The reaction mixture contained 1 μl cDNA, 0.5 μM of each specific primer and 4 μl of Titan Hot Taq Eva-Green Ready Mix in a total volume of 20 μl. The thermal profile was 95°C for 15 minutes, 35 cycles of 94°C for 40 seconds, 60°C for 35 seconds and 72°C for 32 seconds. At the end of each stage, the level of fluorescence emission was obtained for quantification of expression levels. Data were analyzed using the LinRegPCR software version 2012.0 (Amsterdam, Netherland) to obtain the threshold cycle (Ct) and reaction efficiency (28). The transcript level of each target gene relative to *GAPDH* was estimated for each sample in two experimental testes by using efficiency (E) in the formula E_GAPDH_^(Ct sample)^/ E target^(Ct sample)^. The comparison was then statistically analyzed between the two groups of testes. To determine fold change for each gene, the relative gene expression of varicocele-induced testes relative to the non-varicocele testes were calculated as following (29).

Ratio=EGADPH(Ct sample)Etarget(Ct sample)÷EGADPH(Ct control)Etarget(Ct control)

### Statistical analysis

Data are represented as mean ± SE. Differential expression was assessed statistically by using paired t test between the non-varicocele and varicoceleinduced testis pair. When the assumptions behind a parametric test were violated, comparisons were made by the Wilcoxon test. All statistical analyses were performed with the Statistical Package for Social Sciences software version 17 (SPSS Inc., Chicago, IL, USA). When paired t test was done, differences between paired values were consistent and P<0.05 were considered statistically significant.

## Results

### Venographic assessment

**Fig.1:**
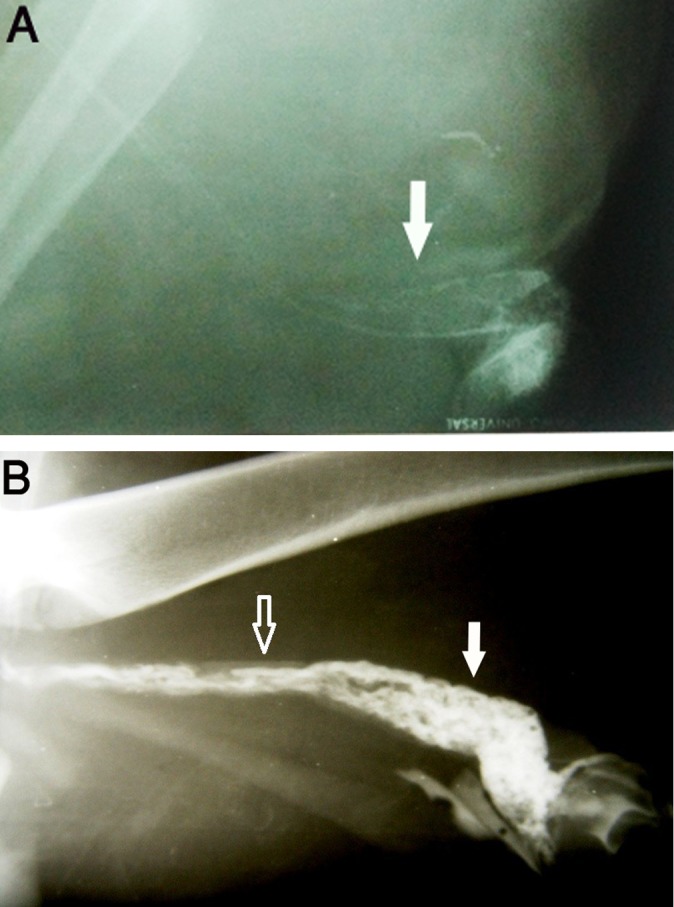
Venography of the right testis in a dog using iohexol contrast media. **A.** Non-varicocele testis; normal circulation in the pumpiniform plexus (white arrow) and **B.** Varicocele-induced testis on the 15^th^ postoperative day; dilatation and congestion in the veins of the pampiniform plexus (white arrow) due to partial occlusion in its proximal part (black arrow).

As observed in the right testicular venogram (Fig .1), dilatation and toruosity of veins of the pampiniform plexus, secondary to retrograde flow, were apparent.

### Histopathologic evaluation

**Fig.2:**
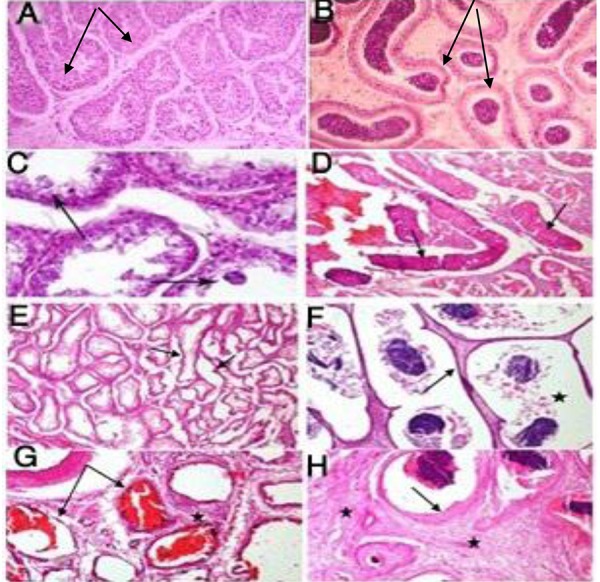
Histopathologic evaluation of varicocele-induced testes after hematoxylin and eosin staining. A. Normal testis with complete spermatogenesis (arrows) (×10), B. Normal epididymis containing spermatozoids and columnar epithelium lining the tubular walls (arrows) (×10), C. Testicular degeneration as spermatogenic arrest at the spermatocyte stage and formation of multinucleated spermatids (arrows) (×40), D. Coagulative necrosis in the seminiferous epithelium (arrows) (×10), E. Testicular atrophy as complete absence of spermatogenesis (arrows) (H<E, ×10), F. Epididimyal epithelial cell pressure (arrows) and dilation of the epididymal tubules (star) (×10), G. Severe congestion (arrows) and dilation of the spermatic cord vessels with inter-vascular fibrosis (star) (×10), and H. Epididymal epithelial metaplasia (arrows) and intertubular fibrosis (star) (×10).

Gross pathologic changes of varicocele-induced testes were congestion, edema and enlargement. Microscopic changes were evaluated after hematoxylin and eosin staining of different sections of testes and were then compared between non-varicocele and varicocele-induced testes. The histopathologic changes consisted of testicular degeneration as well as spermatogenic arrest at the spermatocyte stage and formation of multinucleated spermatid due to failure in spermatid separation (Fig.2A). In addition, coagulative necrosis in the seminiferous epithelium and the presence of eosinophilic material in the seminiferous tubules along with hemorrhage in the interstitium were induced (Fig.2B). Testicular atrophy was also present in the form of complete absence of spermatogenesis (but with normla Sertoli cells) and shrinkage of some seminiferous tubules (Fig.2C). Furthermore, epididimyal atrophy as a prominent dilation of epididimyal tubules with pressure atrophy of their columnar epithelia (Fig.2D), severe congestion and dilation of the spermatic cord vessels with inter-vascular fibrosis (Fig.2E), and epididymal squamous metaplasia and intertubular fibrosis (Fig.2F) were also among the induced histopathologic changes.

**Table 1:** Primers used for reverse transcriptase quantitative polymerase chain reaction (RT-qPCR) of canine transcripts


Target	Sequencing primer (5ˊ-3ˊ)	PCR product (bp)	Accession no.

*GAPDH*	CCCACTCTTCCACCTTCGAC	135	NM_001003142.1
	CCTTGGAGGCCATGTAGACC		
*KITLG*	GGACTTGGAGACGGTGGCAT	130	NM_001012735.1
	CCTAAGGGAGCTGGCTGCAA		
*KIT-receptor*	CAGAGCCCGCAGTAGATTGG	108	AF448148.1
	CAGACGGTGACAGAGACGAG		
*IL-4*	TGGGTCTCACCTCCCAACTG	154	NM_001003159.1
	GTCAGCTCCATGCACGAGTC		
*TNF*	GCCGTCAGATGGGTTGTACC	116	NM_001003244.4
	TCTGGTAGGAGACGGCGAAG		
*HSP90*	TGTGGGAATGACCCGGGAAG	106	AB981782.1
	TGGCCATCCTCTTGTGCCT		
*HSP70*	GACCGCCTTTCGGAAACCTG	200	XM_005627164.1
	GTGTCGGTGAAGGCCACGTA		


**Table 2:** Relative expression of the six genes analyzed in the testis


Gene	Relative gene expression		Ratio (varicocele/non-varicocele)	Pooled SD	P value
	Non-varicocele testis	Varicocele testis			

*KITLG*	0.084	0.0627^*^	83%	0.009	0.047
*KIT- receptor*	0.187	0.131^*^	71%	0.034	0.004
*IL-4*	0.057	0.070	110%	0.027	0.377
*TNF*	0.030	0.020^*^	76%	0.007	0.035
*HSP90*	0.704	0.434^*^	62%	0.054	0.029
*HSP70*	0.010	0.029^*^	290%	0.009	0.018


The Johnson score in the varicocele-induced and non-varicocele testes was 4 (1-8) and 9.6 (9, 10) respectively with the difference being statistically significant (P=0.031).

### Expression analysis of the six related genes

Expression level changes of all six genes were quantified using real time quantitative PCR (RTqPCR) and are shown in Table 2. Expression level of GAPDH was not different in non-varicocele and varicocele-induced testes. Expression of *HSP90, KITLG,* the *KIT-receptor* gene and TNF transcripts in varicocele-induced testes was signiﬁcantly lower than non-varicocele testes (P=0.029, 0.047, 0.004 and 0.035 respectively) with fold-changes of 0.62, 0.83, 0.71 and 0.76 respectively. On the contrary, *HSP70* was signiﬁcantly up-regulated (P=0.018, 2.9 fold-change) in varicocele-induced testes. IL-4 transcript levels did not show differential expression between varicocele-induced and non-varicocele testes (P=0.377).

## Discussion

This study was designed to induce an experimental varicocele model by a simple surgical procedure in dog with minimum invasion and to also investigate the expression of a number of genes involved in varicocele-induced infertility. There are many limitations in the study of varicocele pathophysiology in humans with most studies being non-invasive. In addition, there are other factors such as the status of the varicocele, patient age and level of fertility in the subject population that further hinder the identification of its pathologic basis, thus limiting research on varicocele in humans. Because of these limitations, varicocele has been induced in several species as animal models (7). The induction of varicocele in most animal models involves partially occluding the left renal vein medial near to the kidney. Increased venous pressure proximal to the partial occlusion creates the increased pressure in the left internal spermatic vein, thus resulting in dilatation of the left internal spermatic vein and the pampiniform venous plexus. In all models, a midline abdominal incision must be made from xyphoid to pubis to expose the renal and pelvic vasculature (30). In the present study, the surgical approach was only in the inguinal canal region and contrary to other studies abdominal incision was not made, rendering this method more advantageous. This route was also preferred by the Animal Care Committee and was therefore approved. The histopathologic and venographic evaluations of manipulated testes confirmed the induction of varicocele and subsequent infertility (caused by azoospermia), while the nonvaricocele testis was shown to be slightly influenced as the Jonson score showed values ranging 9-10. This may be due to a transient inflammation in the non-varicocele testis.

Some studies have suggested a relationship between cytokine levels and subfertility. It has been found that concentrations of interleukins such as IL-1, IL-6 and TNF were significantly increased in semen of infertile patients (31). In varicocele, it has been also suggested that expression of IL- 1α and IL-1β, as proinﬂammatory cytokines, were increased. These cytokines in varicocele shift the balance in favor of inﬂammation and immune responses and therefore result in harmful effects in testicular tissue, which may lead to male infertility. In the present study, the expression of IL-4 and *TNF* were evaluated in varicocele. We only observed a significant down-regulation for *TNF* but not for IL-4. It has been shown that IL-4 and TNF play anti-inflammatory and pro-inflammatory roles respectively (32). Previous studies have indicated that the level of TNF or the TNF-related apoptosis-inducing ligand does not change in varicocele (33), however, expression of receptors of the TNF-related apoptosis-inducing ligand were different (34). These reports, nevertheless, evaluated TNF or its receptors at the protein level by ELISA, immunohistochemical and Western blotting techniques, while in our study, expression was evaluated at the transcript level by RTqPCR. It has been shown that post-transcriptional and post-translational factors affecting activity of TNF at both gene and protein levels react to different pathways in varicocele. It must be noted that anti-inflammatory cytokines are able to suppress pro-inflammatory cytokines at both transcriptional and post-transcriptional levels. In fact, the balance between pro-inflammatory and anti-inflammatory cytokines determines the outcome and severity of this disease. Therefore, *TNF* downregulation in our study may be due to the effects of anti-inflammatory cytokines such as IL-4 (35).

Another possibility is that the levels and the subsequent effects of many cytokines alters with varicocele duration. These changes could be to some extent related to the interaction of anti-inflammatory (e.g., IL-4) and pro-inflammatory (e.g., TNF) cytokines in a time-dependent manner. It must be, however, noted that non-varicocele testes probably had a slight inflammation, resulting in an increase in pro-inflammatory cytokines such as TNF.

In the current study, the expression of *KITLG* and the KIT-receptor gene were evaluated. These results, for the first time, demonstrated the downregulation of both genes at the transcript level in varicocele-induced testis. Based on various reports, KITLG/KIT-receptor represent one of the key regulators of testicular formation, development and function since its impairment has been observed in gonadal pathologies including testicular developmental defects, infertility and testicular cancer. Downregulation of KITLG/KIT-receptor has been also observed in oligozoospermia/azoospermia, which is associated with an increase in the germ cell apoptosis process (35, 36). Overall, downregulation of KITLG/KIT-receptor, as reported in here, may be a critical factor in varicocele-mediated infertility. It has been documented that expression of KIT is influenced by various cytokines during inflammation depending on the model or type of the cell system used (18). This effect of cytokines on the KIT system may explain the downregulation of KITLG/KIT-receptor in varicocele observed in this study. Of course, this correlation between the KIT system and cytokines in varicocele needs to be demonstrated more comprehensively since the analysis of only two cytokines (i.e., TNF and IL-4) are insufficient to establish this correlation.

The HSPs are present in spermatocytes during meiosis, participating as an element of the synaptonemal complex, and during the maturation stage of spermiogenesis. We observed a significant increase in *HSP70* expression at the transcript level in the testis with varicocele. In agreement with these results, an increase in HSP proteins has been reported in sperm from oligozoospermic and varicocele individuals (23). Afiyani et al. (37) and Khosravanian et al. (38, 39) have also reported the overexpression of HSP70-2 an HSP2A in varicocele testes. This cellular response is probably an attempt to repair spermatogenic and germ-cell damage due to heat stress. But it must be noted that the expression of all HSP members at the transcript level could not increase during damage of varicocele to protect the testicular cells as we observed here for *HSP90* expression or that observed in Lima et al. (40) who examined the expression of *HSP2A*. Probably, in a time-stage of varicocele, transcriptional apparatus for some of the HSP members would itself be prone to damage. On the other hand, this situation could exacerbate the damage of varicocele in a positive feedback.

## Conclusion

Our data show that partial occlusion of the proximal part of the pampiniform plexus induces varicocele in the testis of dog. The expression of *HSP90, TNF, KITLG *and the* KIT-receptor* gene were considerably decreased in varicocele-induced testes while *HSP70* was increased. *IL-4*, however, did not show differential expression. It is likely that these expression changes may be involved in the pathophysiology of varicocele and related subfertility.

## References

[1] Naughton CK, Nangia AK, Agarwal A (2001). Pathophysiology of varicoceles in male infertility. Hum Reprod Update.

[2] Resim S, Cek M, Fazlioğlu A, Çaşkurlu T, Gürbüz G, Sevin G (1999). Echo-colour Doppler ultrasonography in the diagnosis of varicocele. Int Urol Nephrol.

[3] Gat Y, Bachar GN, Zukerman Z, Belenky A, Gornish M (2004). Varicocele: a bilateral disease. Fertil Steril.

[4] Sandlow J (2004). Pathogenesis and treatment of varicoceles. BMJ.

[5] Redmon JB, Carey P, Pryor JL (2002). Varicocele-the most common cause of male factor infertility. Hum Reprod Update.

[6] Jarow JP (2001). Effects of varicoele on male infertility. Hum Reprod Update.

[7] Salama N, Bergh A, Damber JE (2003). The changes in testicular vascular permeability during progression of the experimental varicocele. Eur Urol.

[8] Sheehan MM, Ramasamy R, Lamb DJ (2014). Molecular mechanisms involved in varicocele-associated infertility. J Assist Reprod Genet.

[9] Hamada A, Esteves SC, Agarwal A (2012). Insight into oxidative stress in varicocele-associated male infertility: part 2. Nat Rev Urol.

[10] Gurdal M, Kirecci S, Huri E, Karaman I, Turkeri L (2008). Correlation between duration of varicocele and apoptosis in testicular tissue in an experimental model. Urology.

[11] Wang YJ, Zhang RQ, Lin YJ, Zhang RG, Zhang WL (2012). Relationship between varicocele and sperm DNA damage and the effect of varicocele repair: a meta-analysis. Reprod Biomed Online.

[12] Saalu LC, Akunna GG, Enye LA, Ogunmodede OS, Akingbade AM (2013). Pathophysiology of varicocele: evidence for oxidative stress as a mechanism pathway. Eur J Anat.

[13] Rossi P, Sette C, Dolci S, Geremia R (2000). Role of c-kit in mammalian spermatogenesis. J Endocrinol Invest.

[14] Roskoski R Jr (2005). Signaling by Kit protein-tyrosine kinase-the stem cell factor receptor. Biochem Biophys Res Commun.

[15] Kazi JU, Vaapil M, Agarwal S, Bracco E, Pahlman S, Ronnstrand L (2013). The tyrosine kinase CSK associates with FLT3 and c-Kit receptors and regulates downstream signaling. Cell Signal.

[16] Reber L, Da Silva CA, Frossard N (2006). Stem cell factor and its receptor c-Kit as targets for inflammatory diseases. Eur J Pharmacol.

[17] Liang J, Wu YL, Chen BJ, Zhang W, Tanaka Y, Sugiyama H (2013). The C-kit receptor-mediated signal transduction and tumor- related diseases. Int J Biol Sci.

[18] Hanada T, Yoshimura A (2002). Regulation of cytokine signaling and inflammation. Cytokine Growth Factor Rev.

[19] Mithraprabhu S, Loveland KL (2009). Control of KIT signalling in male germ cells: what can we learn from other systems?. Reproduction.

[20] Ronnstrand L (2004). Signal transduction via the stem cell factor receptor/c-Kit. Cell Mol Life Sci.

[21] Sahin Z, Celik-Ozenci C, Akkoyunlu G, Korgun ET, Acar N, Erdogru T (2006). Increased expression of interleukin-1alpha and interleukin-1beta is associated with experimental varicocele. Fertil Steril.

[22] Afiyani AA, Deemeh MR, Tavalaee M, Razi M, Bahadorani M, Shokrollahi B (2014). Evaluation of heat-shock protein A2 (HSPA2) in male rats before and after varicocele induction. Mol Reprod Dev.

[23] Ferlin A, Speltra E, Patassini C, Pati M, Garolla A, Caretta N (2010). Heat shock protein and heat shock factor expression in sperm: relation to oligozoospermia and varicocele. J Urol.

[24] Arrabal-Polo MA, Merino-Salas S (2013). Acute left varicocele in an adult. CMAJ.

[25] Dohle GR, Colpi GM, Hargreave TB, Papp GK, Jungwirth A, Weidner W (2005). EAU guidelines on male infertility. Eur Urol.

[26] Hassanpour H, Afzali A, Fatemi Tabatabaie R, Torabi M, Alavi Y (2016). Cardiac renin-angiotensin system (gene expression) and plasma angiotensin II in chickens with T3-induced pulmonary hypertension. Br Poult Sci.

[27] Hassanpour H, Yazdani A, Soreshjani KK, Asgharzadeh S (2009). Evaluation of endothelial and inducible nitric oxide synthase genes expression in the heart of broiler chickens with experimental pulmonary hypertension. Br Poultry Sci.

[28] Ruijter JA, Ramakers C, Hoogaars WM, Karlen Y, Bakker O, Van den Hoff MJ (2009). Amplification efficiency: linking baseline and bias in the analysis of quantitative PCR data. Nucleic Acids Res.

[29] Hassanpour H, Nikoukar Z, Nasiri L, Bahadoran S (2015). Differential gene expression of three nitric oxide synthases is consistent with increased nitric oxide in the hindbrain of broilers with cold-induced pulmonary hypertension. Br Poult Sci.

[30] Najari BB, Li PS, Ramasamy R, Katz M, Sheth S, Robinson B (2014). Microsurgical rat varicocele model. J Urol.

[31] Moretti E, Collodel G, Mazzi L, Campagna M, Iacoponi F, Figura N (2014). Resistin, interleukin-6, tumor necrosis factoralpha, and human semen parameters in the presence of leukocytospermia, smoking habit, and varicocele. Fertil Steril.

[32] Chowdhury TT, Bader DL, Lee DA (2006). Anti-inflammatory effects of IL-4 and dynamic compression in IL-1β stimulated chondrocytes. Biochem Biophys Res Commun.

[33] Celik O, Kutlu O, Tekcan M, Celik-Ozenci C, Koksal IT (2013). Role of TNF-related apoptosis-inducing ligand (TRAIL) in the pathogenesis of varicocele-induced testicular dysfunction. Asian J Androl.

[34] Dinarello CA (2000). Proinflammatory cytokines. Chest.

[35] Mauduit C, Hamamah S, Benahmed M (1999). Stem cell factor/ c-kit system in spermatogenesis. Hum Reprod Update.

[36] Zhang L, Tang J, Haines CJ, Feng HL, Lai L, Teng X (2011). c-kit and its related genes in spermatogonial differentiation. Spermatogenesis.

[37] Afiyani AA, Deemeh MR, Tavalaee M, Razi M, Bahadorani M, Shokrollahi B (2014). Evaluation of heat-shock protein A2 (HSPA2) in male rats before and after varicocele induction. Mol Reprod Dev.

[38] Khosravanian N, Razi M, Farokhi F, Khosravanian H (2014). Testosterone and vitamin E administration up-regulated varicocele-reduced Hsp70-2 protein expression and ameliorated biochemical alterations. J Assist Reprod Genet.

[39] Khosravanian H, Razi M, Farokhi F, Khosravanian N (2015). Simultaneous administration of dexamethasone and vitamin E reversed experimental varicocele-induced impact in testicular tissue in rats; correlation with Hsp70-2 chaperone expression. Int Braz J Urol.

[40] Lima SB, Cenedeze MA, Bertolla RP, Filho PA, Oehninger S, Cedenho AP (2006). Expression of the HSPA2 gene in ejaculated spermatozoa from adolescents with and without varicocele. Fertil Steril.

